# Why Do Quacks Advertise to Regular Physicians?

**Published:** 1913-07

**Authors:** 


### Why Do Quacks Advertise to Regular Physicians?

It is a common occurrence for physicians to receive circulars advertising methods of treatment, offering commissions for reference of cases or simply asking for patients. In certain instances these advertisements are proper enough—or rather, would be if placed in the advertising columns of a medical journal—with the exception of those offering commissions. But, in many instances, these circulars come from the veriest quacks. If it was not for the "Dr." in the address, we might well imagine that physicians' names had been taken from some extra-professional list, but investigation shows that medical lists are designedly followed. We might suggest that the quacks enjoy waving a red rag before a bull for the pleasure of seeing him charge and hearing him snort. Most of us do snort, but the man who paid for the privilege of stirring us up is not at hand to enjoy his joke. Is it possible that there are enough qualified regularly licensed physicians who respond to these circulars?

